# Monthly Summary

**Published:** 1883-02

**Authors:** 


### MONTHLY SUMMARY.

*Profesional Courtesy.*—Will the time ever come when the spirit of jealously and personal feeling shall depart from all professions and every man shall hail every other as brother and friend? What a spectacle is presented to the world in almost every town and city where professional men most do congregate, of that petty strife and bickering spirit that results in a mutual loss of respect and frequently develops into actual hatred.

It is found as often in the dental profession as in any other. Who has known two or three of the leading dentists of a town or city to be on friendly terms? Instead, we all know the common experience, of being forced to listen to slurring and derogatory remarks of one or more of the profession, sometimes of all as a whole, the manifestation of a churlish, uncharitable spirit that savors of anything but "Professional courtesy" or a conformity to the spirit of the "Code of Ethics." Yet these same old stagers who are frequently not on speaking terms with each other, are apt to be most chary of extending a cordial welcome, to the beginner in practice, and watch with jealous eye his every move, and are quick to condemn anything they may consider "unprofessional."

Now this is not as it should be, and while it is an unpleasant truth, yet it should receive consideration, and sober thought from every dentist who loves his profession and is jealous (in the right spirit) of its honor. Let us strive to lay aside our personal feeling more and be governed by that broad spirit of honorable dealing and true courtesy that refuses to speak ill of any man, and especially of a professional brother, to not only do that, but make an effort to be friendly, and extend the fraternal hand to every one. To whom shall the beginner in practice look for encouragement if not to the older members of the profession? Is it apt to increase his respect for the profession at large or for its leaders, when he sees manifested that unprofessional feeling of jealousy so often exhibited? I fear not.

We all can call to mind a few dentists who seem to feel that every other dentist is a natural enemy, to be treated accordingly. However, I am glad to know that their number is not legion. But I appeal for the honor of our calling when I say, let us cultivate more and more the virtues of forbearance, mutual respect and that unity of effort that will place us, as a profession, on somewhat higher ground in respect.—*Dental Headlight.*

---

*Malformation of the Jaws of a Horse.*—Normally the male horse has forty and the female thirty-six teeth. In each jaw there are six molar teeth upon each side. The specimen was interesting for the fact that there were *seven* teeth in each molar arch. On the left side of the head, the molars had, instead of meeting squarely, shut over each other, so that the lower ones, had been bevelled off upon the outside, and the upper ones upon the inside. On the right side of the head the molars presented the normal appearance, except with reference to number. Some eight or ten years ago, the animal was under his observation, and at that time had difficulty in masticating. Subsequently symptoms of apparent glanders developed, and finally the food passed out through the nostrils. At the post-mortem there was found, besides the deformity of the jaws, disease of the palate bone, that accounted for the fetid discharge and apparent glanders. There was also a marked change in the curve of the incisor arch, so much so, that the lower front teeth were nearly horizonta.l—*Dr. Liautard in Med. Record.*

---

*To Disguise the Odor of Iodiform.*—We have had many queries as to how this may best be accomplished; we therefore give every reliable report on the subject. The following is from the *New York Medical Journal and Obstetrical Review:* "Having tried nearly all the devices that have been suggested for mitigating or disguising the odor of iodiform, and found them all of little or no avail, we have lately come nearer to the object by using oil of eucalyptus according to the following formula :

℞. Pulv. iodiform, ℥ss

Eucalypt., ℥ss

Vaselin, ℥iv.

M. ft. ungent.

This ointment is not without odor, but the odor is not that of iodiform."—*Med. and Surg. Reporter.*

---

*Decolorization of Hair Associated with Neuralgia.*—The *Medical Times and Gazette* says that M. Raymond relates the case of a lady of 38, who was suffering from neuralgia of the scalp. After suffering several days the pain became much worse one evening, and morphia was powerless to relieve it. At two o'clock the next morning the pain was at its worst. At this moment the hair had its normal color ; at seven o'clock the same morning it was found that her hair was almost completely decolorized." It is remarkable that at first the greater part of the patient's hair became red, turning to white a few days later ; and later still, falling off in considerable proportion. The case affords an absolute contradiction to Kaposi's theoretical view that blanching of the hair can never take place very rapidly, but *must* require several weeks for its completion.

---

*Excision of the Tongue.*—Mr. Croly, of Dublin, performs the following operation for cancer of the tongue, even when the disease is situated in the anterior portion of the organ. He first ligatures each lingual artery close to the hyoid bone, through a curved incision, reaching from the symphysis down to the hyoid bone, and up and back to the angle of the jaw. Through these incisions he withdraws the tongue, as in Regnoli's operation, and removes the requisite amount of it by the benzoline cautery. Lastly, he divides the gustatory nerve where it lies along the inner border of the jawbone.—*Med. and Surg. Reporter.*

